# Genetic and Epigenetic Alterations in Pancreatic Carcinogenesis

**DOI:** 10.2174/138920211794520132

**Published:** 2011-03

**Authors:** Yannick Delpu, Naïma Hanoun, Hubert Lulka, Flavie Sicard, Janick Selves, Louis Buscail, Jérôme Torrisani, Pierre Cordelier

**Affiliations:** Inserm UMR 1037- University of Toulouse III, Cancer Research Center of Toulouse (CRCT), BP 84225, CHU Rangeuil, Toulouse 31432, Cedex 4, France

**Keywords:** Pancreas, Cancer, Preneoplastic lesions, DNA methylation, Genetic alteration.

## Abstract

Pancreatic ductal adenocarcinoma (PDAC) is one of the most lethal cancers worldwide. Despite significant progresses in the last decades, the origin of this cancer remains unclear and no efficient therapy exists. PDAC does not arise *de novo*: three remarkable different types of pancreatic lesions can evolve towards pancreatic cancer. These precursor lesions include: Pancreatic intraepithelial neoplasia (PanIN) that are microscopic lesions of the pancreas, Intraductal Papillary Mucinous Neoplasms (IPMN) and Mucinous Cystic Neoplasms (MCN) that are both macroscopic lesions. However, the cellular origin of these lesions is still a matter of debate.

Classically, neoplasm initiation or progression is driven by several genetic and epigenetic alterations. The aim of this review is to assemble the current information on genetic mutations and epigenetic disorders that affect genes during pancreatic carcinogenesis. We will further discuss the interest of the genetic and epigenetic alterations for the diagnosis and prognosis of PDAC. Large genetic alterations (chromosomal deletion/amplification) and single point mutations are well described for carcinogenesis inducers. Mutations classically occur within key regions of the genome. Consequences are various and include activation of mitogenic pathways or silencing of apoptotic processes. Alterations of K-RAS, P16 and DPC4 genes are frequently observed in PDAC samples and have been described to arise gradually during carcinogenesis. DNA methylation is an epigenetic process involved in imprinting and X chromosome inactivation. Alteration of DNA methylation patterns leads to deregulation of gene expression, in the absence of mutation. Both genetic and epigenetic events influence genes and non-coding RNA expression, with dramatic effects on proliferation, survival and invasion. Besides improvement in our fundamental understanding of PDAC development, highlighting the molecular alterations that occur in pancreatic carcinogenesis could provide new clinical tools for early diagnosis of PDAC and the molecular basis for the development of new effective therapies.

## 1 INTRODUCTION

Pancreatic ductal adenocarcinoma (PDAC) is the fourth cause of death by cancer in Western countries, while representing only 3% of the new cancer cases each year [1]. Tobacco, diabetes, obesity and chronic pancreatitis are described as risk factors for PDAC [2]. However, with the exception of familial PDAC, no high risk population is defined. Consequently, screening for PDAC with current imaging techniques seems possible only for patients presenting a familial history of PDAC or already managed for others pancreatic diseases. Hence, PDAC is diagnosed at late stage due to the absence of specific symptoms (abdominal discomfort and abdominal mass due to compression of pancreatic duct) [2, 3]. Up to 60% of patients have advanced PDAC at the time of diagnosis and are not eligible for surgery; their median survival is only 3 to 6 months [1]. Resection relies on the general condition of the patient, the location, size and tumor local invasion. Because of its poor prognosis, improvement of PDAC prognostic requires detecting and managing this cancer before regional invasion and metastasis [3]. For a better clinical care and a better understanding of PDAC, remarkable works have been done to elucidate the early events driving pancreatic carcinogenesis. We will first describe the main preneoplastic lesions leading to PDAC, to further introduce the molecular alterations associated with PDAC and their lesion-stage dependent appearance. We will finally discuss the molecular marker potential and the clinical interest of such alterations.

## 2 PRENEOPLASTIC LESIONS OF THE PANCREAS

To date, three types of neoplasic lesion precursor to PDAC are described and characterized. The study of these lesions is of prime importance to:

1. detect and manage PDAC before it becomes invasive and incurable.
2. better understand PDAC carcinogenesis to elaborate new therapies.

Development of such lesions is most of the time asymptomatic, and their discovery occurs together with unresecable carcinoma. They all follow a multistep progression to invasive cancer classified upon morphological and cytological features [2]. The sequential evolution from precursor lesion to cancer is still a matter of debate because of the absence of early detection and follow-up of these lesions. Main obstacles for precursor detection and follow-up using current diagnostic technologies are their small size and the absence of high risk population to screen. Consequently, studies are usually conducted on fixed resected tissue. In other words, the following observations are in favor of a multistep carcinogenesis:

1. duct lesions are far more common in pancreas with infiltrating carcinoma [4, 5].
2. there is an increase in the grade of lesions surrounding infiltrating carcinoma [5].
3. patients diagnosed with intraductal mucinous neoplasms that are not resected eventually develop infiltrating carcinoma [4, 5].

### 2.1 Pancreatic Intraepithelial Neoplasia (PanIN)

**Fig. (1):**
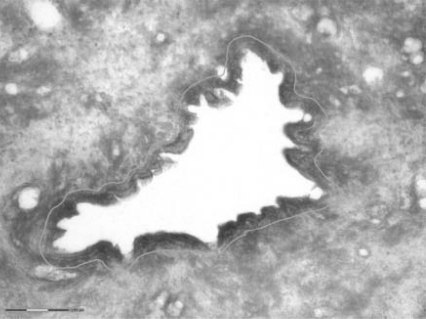
Microscopic view of Pancreatic intraepithelial neoplasia.

PanINs are microscopic ductal lesions usually measuring less than 1 cm, generally located in the head of the pancreas. The small size of PanINs renders illusive their detection by MRI (Magnetic Resonance Imagery) or CT (Computed Tomography). Observation of these lesions is usually fortuitous and concomitant to tumor resection. PanINs are classified into 3 grades: PanIN-1 (which are subdivided into PanIN-1A and PanIN-1B), PanIN-2 and PanIN-3/ *in situ *carcinoma (Fig. **1**). This classification depends on the degree of architectural and cytological atypia. Briefly, flat and normal epithelia of secondary duct branches show increasing papillary architecture and finally adopt a severe cellular atypia and frequent mitosis, preceding invasive carcinoma [2]. PanINs are the most frequent preneoplastic lesions of the pancreas and are observed in 82% of pancreas with pancreatic cancer [5]. Low grade PanINs appear to be common lesions found in 16-80% of normal pancreas in the absence of neoplasia, while high grade PanINs are nearly exclusively associated with adenocarcinoma [5-8]. Besides, it is suggested that only 1% of PanINs evolve into PDAC [9]. As a result, it is tempting to consider low grade PanINs as benign lesions in absence of key point mutations. Key genetic events would enhance PanIN-1 to PanIN-2 transition, leading to a “no turning back” development into PDAC. A mouse model expressing the constitutively activated K-RAS G12D proto-oncogene recapitulates PanIN lesions and is the common *in vivo* model for PanINs study [10]. 

### 2.2 Intraductal Papillary Mucinous Neoplasms (IPMN)

**Fig. (2):**
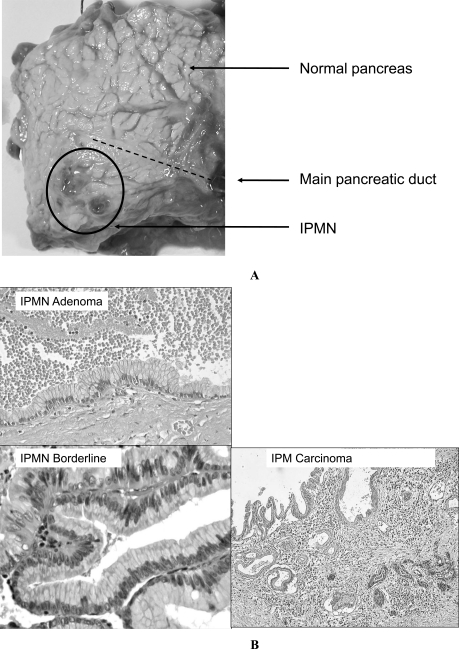
**A:** Macroscopic view of an Intraductal Papillary Mucinous Neoplasm. **B:** Microscopic view of an Intraductal Papillary Mucinous Neoplasm.

IPMNs are far less common than PanIN lesions and account for 3-5% of pancreatic tumors [11]. IPMNs are divided into adenoma, borderline and intraductal papillary mucinous carcinoma (IPMC) according to the highest degree of dysplasia detected (Fig. **2A** and **2B**) [12]. They comprise large lesions with long finger-like papillary architecture, larger than 1 cm and delimited by a columnar mucin-secreting epithelium [13, 14]. IPMNs communicate with pancreatic main duct. Invasive carcinoma occurs in 20-50% of IPMNs and are located on the main pancreatic duct or in the secondary branches of the main duct [11]. Additionally, further anatomical criteria depending on the extension of the disease in the organ are taken into consideration. In fact, IPMNs are classified into three groups: main pancreatic duct IPMN (MPD-IPMN), branch duct IPMN (BD-IPMN), and mixed IPMN [15]. Current knowledge on IPMN indicates that approximately 70% of MPD-IPMNs may progress into invasive cancer [15]. Resection represents the only curative treatment for IPMNs [15]. The long-term follow-up of resected patients shows 5-year survival rates of 88% for benign and non-invasive lesions and between 36 and 60% for IPMC [15, 16]. 

PanINs and IPMNs lesions exhibit similar histological and molecular features and the distinction between those two types of lesions is based on their size and location. Depending on the lesion origin (main duct or peripheral ductules), PanINs and IPMNs can be considered as similar lesions with different clinical outcome. Moreover, a mouse model develops PanIN lesions in addition to cystic lesions of the pancreas resembling human IPMNs following TGF-α overexpression in mutant K-RAS(G12D) transgenic mice [17]. This observation is in favor of a common origin of both types of lesions.

### 2.3 Mucinous Cystic Neoplasms (MCN)

**Fig. (3):**
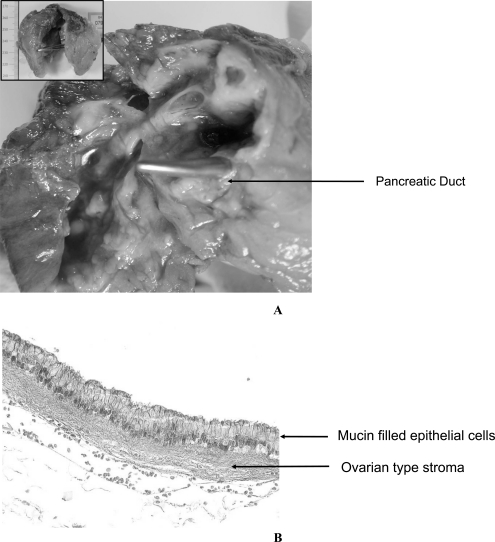
**A:** Macroscopic view of Mucinous Cystic Neoplasm. **B:** Microscopic view of Mucinous Cystic Neoplasm.

MCNs are rare and large (1 to 3 cm) mucin secreting neoplasms [13]. This type of lesions is lined by a columnar, mucin-filled epithelium and shows a characteristic ovarian-type stroma beneath the epithelial layer, separating the lesion from the pancreatic main duct (Fig. **3A** and **3B**). MCNs present a higher incidence in women as compared to men (ratio 20 to 1) for unknown reasons. MCNs are classified according to their degree of epithelial dysplasia: Mucinous cystadenoma, borderline mucinous cystic neoplasms and mucinous cystic neoplasms with *in situ* carcinoma [18]. Because of their size and their location, they are often associated with non-specific symptoms (abdominal discomfort and abdominal mass due to compression of pancreatic duct). About 20% of MCNs are associated with PDAC [11]. All MCNs may progress to mucinous cystadenocarcinoma, which has a very low resectability and a very poor prognosis [35, 36, 15]. The 5 year-survival rate for patient with a surgically resected MCNs in the absence of PDAC is close to 100%; such survival rate falls to 60% when an adjacent neoplasia is diagnosed [19]. Izeradjene and co-workers demonstrated that mice expressing activated K-RAS(G12D) and deleted for Smad4/Dpc4 develop pancreatic cystic neoplasms in the body and the tail of the pancreas in addition to low-grade PanINs [20]. According to the size of these lesions, IPMNs and MCNs can be detected using CT and MRI and present a better prognostic as compared to microscopic lesions PanIN.

## 3 CELLULAR ORIGIN OF PRENEOPLASTIC LESIONS

All three known precursor lesions bear ductal epithelial cells characteristics, but the precise cellular origin of these lesions is still highly debated. 

In mice, induction of short term pancreatic injuries using caerulein leads to transient acinar cells dedifferentiation into mesenchymal cells. These proliferating cells differentiate into acinar cells to partially repopulate the wounded organ [21]. In mice carrying activating K-RAS mutation, this process seems altered and mesenchymal cells differentiation is impaired, leading to acinar-to-ductal metaplasia, and then to PDAC precursor lesions such as PanINs [21]. The contribution of immature pancreatic precursors to PDAC progression raises the possibility of the presence of pancreatic cancer stem cells (CSC). Classically, stem cells constitute a rare cellular population characterized by its self-renewal, specific cell surface markers, and cellular efflux through multidrug resistance channels. Furthermore, these cells give rise to an heterogeneous lineage [22-25]. Cancer stem cells may derive from this particular population, to acquire a slightly transformed phenotype, known as “minimal deviation” [23]. 

CSCs have been identified in breast cancer, myeloid leukemia, colon and prostate cancer [26]. The identification of such CSCs in the pancreas only relies on the presence of cell surface markers and the up-regulation of Sonic Hedgehog pathway (SHH), participating in self renewal mechanisms [27]. Zhou and co-workers reported dye efflux capacities of PANC-1 cells side population *in vitro* but not *in vivo *[22, 28]. If the capacity of efflux, the location and the rarity of these cells within the tumor would contribute to explain their resistance against conventional therapies, this evidence only suggests the “stem” nature of a subpopulation of pancreatic cancer cells and further studies will be required to conclude on their status. Furthermore, the existence of pancreatic cancer stem cells implies the existence of adult pancreatic stem cells to deviate from. To date, existence of such cell type is still a matter of debate [21, 29]. On the other hand, one could hypothesize that a so-called stem cell population would rather arise from a subpopulation of de-differentiated acinar cell, unable to fully differentiate into epithelial ductal cells. Additionally, adult stem cell performing minimal deviation and leading irremediably to PDAC is contradictory because of the frequent observation of low grade PanINs in normal pancreas. “Cancer stem cell” would then originate from dedifferentiation failure during pancreas regeneration. The status of mesenchymal cell would allow them to migrate, colonize new niche and then, enter dormancy. Consequently, residual undifferentiated pancreatic cell in resection margin would explain frequent relapse of patients after surgery, together with distant, microscopic metastasis.

## 4 DNA ALTERATIONS IN PRENEOPLASTIC LESIONS

Classically, sequential evolution from precursor lesion to PDAC involves the accumulation of diverse molecular changes. Such alterations provide precious evidence for understanding PDAC development, and devising new detection and diagnostic tools. However, little is known about the kinetic of appearance of these molecular events. To date improvement in high throughput sequencing technologies provides whole genomic methylation patterns and single point mutation analysis. Moreover, recent design of mouse models recapitulating preneoplastic lesions of the pancreas allowed great advances in our understanding of PDAC related molecular alterations. 

### 4.1 Genetic Alterations

#### 4.1.1 Large Chromosomal Alterations in PDAC

Genetic alterations in pancreatic cancer have been described for decades. In the late 80’s, Korc and coworkers reported that Epidermal Growth Factor Receptor (EGFR) altered expression is linked with qualitative and quantitative chromosomal defects such as reciprocal translocation and chromosomal deletion of chromosome 7 [30]. EGFR belongs to the cell surface receptor family with tyrosine kinase activity. EGFR activation by its ligand EGF leads to signal transduction through three main pathways: Mitogen-activated protein (MAP) kinase cascade, phosphotidylinositol-3 kinase (PI3K) pathway and the signal transducer and activator of transcription (STAT) pathway, promoting cell division and survival [31]. In PDAC as in other cancers, EGFR is frequently subjected to gene amplification (65% of cases) [32-34]. Intragenic mutations are far less common (3.6% of PDAC) and lead to EGFR over-activation [35]. Overexpression of EGFR is an early event in pancreatic carcinogenesis and occurs from PanIN-1 stage to invasive PDAC [36]. Besides, 61.2% of malignant MCNs exhibit increased expression of EGFR, whereas EGFR is not detected in any of benign MCN [37]. 

Recent studies based on high throughput sequencing approaches described numerous original regions prone to frequent large genetic alterations during carcinogenesis. One hundred and forty four minimal regions identified in 119 independent loci are subjected to such changes and play a potential role in tumor progression, with loci encoding for p16INK4A, TP53, MYC, K-RAS2, and AKT2 previously described as duplicated or deleted in PDAC [38]. Using Representational Oligonucleotide Microarray (ROMA), Lucito *et al.* identified 31 amplifications and 25 deletions involving more than 500 genes in familial pancreatic cancer [39]. Campbell *et al.* recently analyzed patterns of genomic instability in PDAC and pancreatic metastasis [40]. Resulting evidence indicates that PDAC bears specific pattern of genomic alterations (deletion, fold-back inversion, tandem duplication), as compared to other type of cancer. Moreover, these alterations are sequentially detectable from primary tumor to metastasic disease. However, the status of such alterations in preneoplastic lesions is unknown. Additionally to tumor suppressor genes, genetic instability occurs in specific regions encoding microRNAs [41]. MicroRNAs (miRNA) are small non coding RNAs (20-22nt) involved in the negative regulation of mRNAs translation [42]. miRNAs may regulate about 60% of cellular processes and their deregulation is a critical event in carcinogenesis. Alteration in miRNAs expression is a hallmark of cancer and leads to oncogene loss of repression or direct tumor suppressor gene downregulation [42]. Such deregulation leads to a wide range of consequences, influencing tumor progression including cell cycle regulation, epithelial to mesenchymal transition, cellular migration and angiogenesis. Over 130 microRNAs are documented as deregulated in PDAC and pancreatic cancer cell lines but few data exist on the precise mechanisms of such alteration [43, 44]. Similarly to tumor suppressor genes, miRNAs expression alteration can occur anywhere from early to late precursor lesions during PDAC progression. In fact 52.5% of miR genes are in cancer-associated genomic regions or in fragile sites, subjected to loss of heterozygosity, minimal regions of amplification, or common breakpoint regions [45]. 

#### 4.1.2 Single Point Mutation 

Single point mutations correspond to single base substitution in DNA sequence. Such replacement can have various outcomes depending on the genomic region targeted and the new codon generated by this modification. Many tumor suppressor genes and proto-oncogenes are associated with activating or inhibiting mutation in PDAC. If many of these mutations are known for decades, the dynamic of their appearence in preneoplastic lesions of the pancreas have long remained unclear. Here, we present a non-exhaustive list of genes for which single point mutation have been identified in preneoplastic grades. The **K-RAS** gene (Kirsten-RAS) encodes a RAS homolog that belongs to the cytosolic GTPase family. A single amino acid substitution from G to D at codon 12 is responsible for a constitutive activation observed in up to 95% (60-95%) of PDAC and therefore represents the most common mutation for this cancer [46]. Oncogenic K-RAS activates the MAP Kinase, and/or the PI3K pathways, leading to increased mitogenic activity. K-RAS mutation is an early event during pancreatic carcinogenesis and occurs in 35% of PanIN-1 lesions and reaches 75% in PanIN-3 [6, 46]. The low frequency of K-RAS mutation in PanIN-1 suggests that K-RAS mutation facilitates the progression from low-grade to high-grade PanINs but is not a *sine qua non* condition to PanIN-1 initiation. On the other hand, K-RAS G12D mutation specifically expressed in the whole mice pancreas at the embryonic stage is sufficient to develop PanIN lesions [10, 47]. K-RAS mutation is observed in a third of IPMN adenomas and its mutation frequency increases with lesion grade (50 % of mutated K-RAS in IPMN borderline and IPMC with invasion) [12, 48]. Similarly, MCNs harbor K-RAS mutation in 20% of benign MCNs, 33% of borderline lesions, and 89% of malignant MCNs [49]. **B-RAF** is a member of the RAF family, belonging to the MAP Kinase pathway. B-RAF mutation is observed in 7%-15% of PDAC [12, 50]. Activating intragenic mutation of B-RAF leads to activation of RAF effector MEK. Previous reports proposed that K-RAS and BRAF mutation are exclusive in PDAC [2, 51] and that oncogenic B-RAF would represent an alternative for MAP Kinase pathway activation in the absence of K-RAS mutation [50]. Ishimura and co-workers reported concomitant K-RAS and BRAF activating mutations in tumor samples [50], with no clinicopathological incidence as compared to K-RAS mutation alone, suggesting that there is no cumulative effect and that the addition of the two mutations is not an advantage for tumor progression. When compared to the very high K-RAS mutation frequency in preneoplastic lesions, mutation of B-Raf only occurs in 2.7% of IPMTs [12]. The study of B-RAF mutation in PanINs and MCNs is still to be done. As mentioned above, Schonleben *et al *reported that **EGFR** mutations are very infrequent in IPMNs (no mutation found in 36 IPMT) whereas another group reported EGFR point mutation in 7.5% of Intestine Type IPMNs [48, 52]. To date, no mutational analysis of EGFR concerning PanIN and MCN lesions are available. ***DKN2A*** is a tumor suppressor gene which encodes the P16 tumor suppressor protein. P16 indirectly provokes Rb protein phosphorylation and its loss of function leads to entry in cell cycle and acute cell proliferation. P16 is the most frequently inactivated tumor suppressor gene in PDAC and is observed in up to 95% of patients [13]. Interestingly, different mechanisms are responsible of its inactivation including homozygous deletion, intragenic mutation and epigenetic silencing by promoter methylation (40%, 40% and 15% of PDAC respectively) (see paragraph below) [2]. All of these invalidating modifications arise at late stages of pancreatic carcinogenesis, in both PanINs and IPMNs (adenoma 45%, borderline 50%, IPMC 53%) with a greater incidence in PanINs (PanIN-1 30-44%, PanIN-2 80%, PanIN-3 92-100%) [53]. However, these data are based on protein expression studies using immunohistochemistry detection and does not identify the alterations leading to p16 loss of function. Cell line derived from non invasive MCNs harbors p16 mutation at codon 58, indicating that such genetic alteration is an early event in MCNs multistep development [54]. **SMAD4/DPC4** (Mothers against decapentaplegic homolog 4/Deleted in Pancreatic Cancer 4) is subjected to frequent deletion or intragenic mutation in PDAC (55%) [55]. SMAD4 is a key actor in TGF beta signaling pathway, and activates transcription of cell cycle inhibitory factors, particularly p21 [56]. Thus, loss of SMAD4 leads to relapse of growth inhibition and uncontrolled proliferation [53]. SMAD4 expression is normal in low grade PanINs (PanIN-1 and -2) and inactivation occurs at late stage in 31% of PanIN-3 [55]. Similarly, SMAD4 expression is normal in adenoma and borderline IPMNs and occurs in 38% of IPMCs [53]. During MCNs development, DPC4 loss of expression is a late event, as the protein is not detectable at invasive stage [57]. 

Mutation in Tumor Protein 53 (**TP53**) locus is the most common single point mutation in human cancer. TP53 acts as a transcription factor and binds to DNA to activate transcription of genes regulating DNA repair, cell cycle and apoptosis [56]. Inactivating mutations in TP53 are observed in up to 75% of pancreatic cancers [13]. Nonetheless, mutational inactivation of TP53 is a late event during PanIN multistep progression to PDAC (0% in PanIN-1 to 12% in PanIN-3) and seems to be more frequent in IPMN (30% in IPMN adenoma, 30% in IPMN borderline and in IPMC 50-63%) [2, 13, 53, 56, 58]. Cell line derived from non invasive MCN harbors p53 mutation at codon 132, indicating that such genetic alteration takes place before invasive MCN grade [54]. Whole genome analysis approach represent the next step for the discovery of new actors that have not previously been linked to cancer progression or initiation. The genome-wide study of twelve prospective cohorts of PDAC patients identified SNPs located in three novel genomic regions associated with the risk of PDAC [59]. Two of these regions are coding region while the third locus on chromosome 13q22.1 maps to a large non coding region that requires further characterization.

### 4.2 Epigenetic Alterations

**DNA methylation** consists of the addition of a methyl (CH3) residue on cytosine preceding a guanosine, known as CpG dinucleotides (p stands for phosphate). This modification is catalyzed by a DNA methyltransferase family (DNMT1, 3a and 3b). CpG dinucleotides are not equally interspaced in the genome and are frequently grouped in CpG island. Sixty percent of gene in human genome display one or more CpG islands in their promoter and therefore can be potentially inactivated by methylation. Only 5% of these promoters are methylated. They promote transcription of genes involved in development , or subject to X chromosome inactivation and genomic imprinting [60]. Alteration in DNA methylation pattern is a hallmark of cancer and is associated with an overexpression of DNMTs [61, 62]. Interestingly, whole genome analysis indicates global hypomethylation and only local hypermethylation [63, 64]. Global hypomethylation phenomenon despite DNMTs overexpression may involve targeted DNA methylation [65]. However, the molecular mechanisms involved in such regulation remain unclear to date. Frequent over expression of proto-oncogene in cancer may be linked to their genomic sequence hypomethylation. Understanding global methylation patterns has long been limited by technological concerns limiting the number of genes analyzed. Using cell lines and tumor samples, Sato and co-workers analyzed the methylation status of a subset of 18 genes previously identified as over expressed in PDAC as compared to normal pancreas [66]. The authors describe that Mesothelin and Claudin 4 genes are frequently hypomethylated in PDAC (92% and 89%, respectively) and their methylation status correlates with their expression pattern. On the other hand, hypermethylation occurs during cancer, and may lead to gene silencing. Hypermethylation is responsible for the inactivation of numerous tumor suppressor genes and can occur independently or additionally to intragenic mutation [66-68]. As an example, P16 is equally subjected to mutation or hypermethylation, whereas RASSF1A, Cyclin D2, SOCS-1, APC are tumor suppressor genes silenced only following hypermethylation of their promoting sequence in PDAC [69-71]. By comparing the whole methylome of PDAC with healthy tissue using high throughput technologies, Omura *et al. *determined that MDF1, miR-9-1, ZNF415, CNTNAP2 and EVOLV-4 were the most frequently methylated loci among 88000 probes tested [68]. Interestingly, only miR-9-1 and CNTNAP2 have previously been linked to cancer progression or initiation. In PanINs, eight genes (*CDH3, reprimo, CLDN5, LHX1, NPTX2, SARP2, SPARC *and* ST14*) were reported to be differentially methylated between normal epithelia and PanIN lesions [72]. Interestingly the pattern of methylation during the progression of PanINs is variable among genes. This work proves that CpG island hypermethylation is an early event in PDAC initiation, and its prevalence progressively increases during neoplastic progression. Similar results were found in IPMNs concerning another subset of genes including P16, ppENK, P14, P15, P17, APC, hMLH, E-Cadherin and MGMT [73]. Gene methylation status have not been studied yet in MCN. Taken together, DNA aberrant hypermethylation correlates with DNMT1 over expression in PanINs, and strongly suggests that early methylation may induce precursor lesion progression, and is probably not a consequence of deregulated cellular processes [62]. As stated before, **miRNAs **have been linked to cancer initiation or progression but miR-148a, 107, 34a and Mir 9-3 are the only miRNA genes hypermethylated in cancer [41, 74-76]. Few miRNAs are described as early actors of pancreatic carcinogenesis. MiR-148a is described to be down regulated in numerous cancers (colon, melanoma, lung cancer) [41]. Our recent works demonstrated mir-148a down regulation by DNA hypermethylation in PanIN-1 and -2 [74]. To date, no data are available concerning miR-148a expression in IPMNs or MCNs. On the other hand, miR-200 and miR-34a alteration of expression are late events in PDAC. Indeed, miR-200 overexpression takes place in invasive PDAC consequently to its genomic sequence hypomethylation and indirectly suppresses E-cadherin expression and contributes to epithelial to mesenchymal transition [77]. Inversely, miR-34a is down-regulated by hypermethylation of its DNA coding sequence and its re-expression induces senescence and cell cycle arrest [76, 78]. Consistent work has been done on molecular characteristics of invasive cancer. Further efforts will be needed to improve our understanding of molecular alterations in preneoplactic lesion during their multistep evolution. In fact, most of the studies are performed on PanIN lesions, due to their higher frequency as compared to IPMNs and MCNs. Further work is needed to better understand IPMN and MCN carcinogenesis. As described above, alteration in miRNAs expression caused by aberrant DNA methylation is an early event in PDAC carcinogenesis, but the consequences of such modification is not clearly defined. Because miRNAs are fine regulator of multiple cellular pathways and because their alteration occurs very early in the oncogenic cascade, it is tempting to speculate that miRNAs may induce carcinogenesis. Transgenic mouse models for miR-17−92, miR-21, miR-29 or miR-155 miRNAs were recently published [79-82]. In these models, expression of miR specifically targeted to the hematopoietic compartment induced lymphopathies. To date, no PDAC transgenic mouse model with impaired miRNAs expression is available. 

## 5 GENERAL INTEREST OF GENETIC MUTATION AND METHYLATED GENE AS BIOMARKERS FOR PDAC

For routine clinical use, molecular markers must be highly sensitive, highly specific and easy to access. To date, current molecular markers mainly rely on variations of proteins/mRNAs expression or histological features. However, the use of such markers presents several issues: i: proteins and mRNAs are not stable and are subject to extensive degradation following collection and ii: histological and protein analysis require consequent amount of tissue. PDAC grading is currently based on CA 19-9, mucin expression or histological study from pancreatic fluids or biopsy. Determination of new molecular markers to improve early diagnosis of this malignancy is impeded by the low frequency of PDAC (4% of new cases of cancer) and its late diagnosis [1]. Genetic and epigenetic alterations both represent an important source of biomarkers for PDAC early detection. In comparison to standard molecular markers, DNA mutation and DNA methylation patterns confer the advantage to be stable and quantified from very low amounts of material from diverse biological samples (biopsies, urines, stool, blood…).

### 5.1 Diagnostic Marker

Recent studies considered K-RAS mutation analysis in Fine Needle Aspiration (FNA) for PDAC diagnosis, and achieved respectable sensitivity and specificity [83-85]. Our work indicates that miR-148a genomic sequence hypermethylation also discriminate PDAC from chronic pancreatitis [74]. Moreover, 289 methylation patterns in 2016 genes have been demonstrated to successfully discriminate healthy tissue from PDAC [67]. However, this pattern is not specific for PDAC among other types of cancer and requires biopsy so is not suitable for large scale screening of PDAC. Finally, recent works proved that measuring methylation patterns specific for PDAC in blood samples is possible with a sensitivity of 81.7%, and can be a first step towards a sensitive, specific and non-invasive diagnosis tool [86]. 

#### 5.1.1 Predictive Marker

Another promising application of genetic mutation and methylated gene derived biomarkers is to predict patients resistance to therapy. According to Tan and co-workers, methylation status of GSTM1 and ONECUT2_E96_F is predictive of tumor chemosensitivity [67]. Determination by high throughput technologies of global methylation patterns in patients diagnosed for PDAC and correlation with response to gemcitabine treatment after follow up could identify predictive and surrogate markers for chemotherapeutic response [67].

## 6 Summary and future directions

Despite recent progress in understanding the multistep progression from precursor lesion to invasive cancer and metastasis, PDAC is still detected too late for surgical treatment. As a consequence, early diagnosis of this disease is urgently needed. Detecting alterations in DNA methylation patterns is one most promising avenue for biomarker discovery for PDAC. It is our belief that the future use of unbiased high throughput sequencing will allow the characterization of whole methylation patterns to identify new biomarkers helpful for PDAC diagnosis and prognosis.
